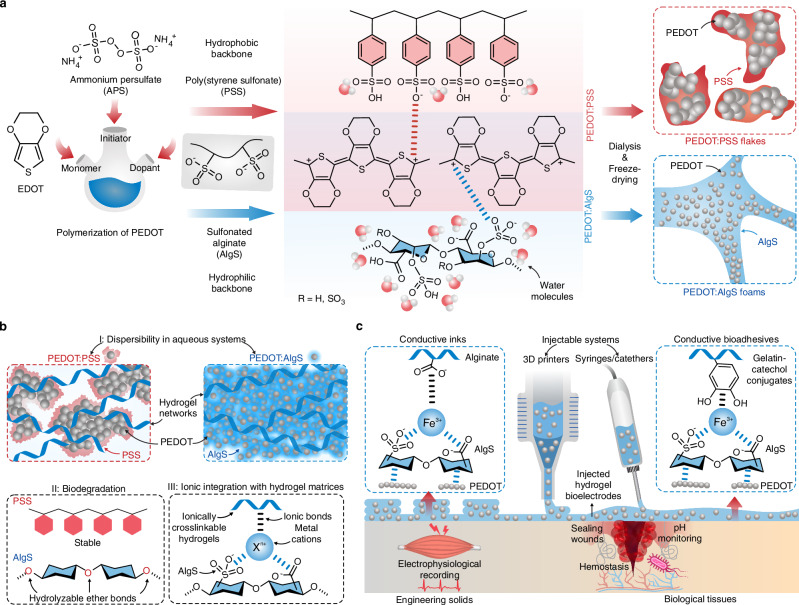# Author Correction: Boosting hydrogel conductivity via water-dispersible conducting polymers for injectable bioelectronics

**DOI:** 10.1038/s41467-025-60718-0

**Published:** 2025-06-12

**Authors:** Hossein Montazerian, Elham Davoodi, Canran Wang, Farnaz Lorestani, Jiahong Li, Reihaneh Haghniaz, Rohan R. Sampath, Neda Mohaghegh, Safoora Khosravi, Fatemeh Zehtabi, Yichao Zhao, Negar Hosseinzadeh, Tianhan Liu, Tzung K. Hsiai, Alireza Hassani Najafabadi, Robert Langer, Daniel G. Anderson, Paul S. Weiss, Ali Khademhosseini, Wei Gao

**Affiliations:** 1 David H. Koch Institute for Integrative Cancer Research, Massachusetts Institute of Technology, Cambridge, Massachusetts USA; 2 Department of Bioengineering, University of California, Los Angeles, Los Angeles, California USA; 3 Mechanical Engineering Department, University of Utah, Salt Lake City, Utah USA; 4 Terasaki Institute for Biomedical Innovation, Los Angeles, California USA; 5 Andrew and Peggy Cherng Department of Medical Engineering, Division of Engineering and Applied Science, California Institute of Technology, Pasadena, California USA; 6 Department of Engineering Science and Mechanics, Pennsylvania State University, University Park, Pennsylvania, USA; 7 Department of Chemistry and Biochemistry, University of California, Los Angeles, Los Angeles, California USA; 8 Department of Electrical and Computer Engineering, University of British Columbia, Vancouver, British Columbia Canada; 9 Department of Chemical Engineering, Massachusetts Institute of Technology, Cambridge, Massachusetts USA; 10 Department of Anesthesiology, Boston Children’s Hospital, Boston, Massachusetts USA; 11 Institute for Medical Engineering and Science, Massachusetts Institute of Technology, Cambridge, Massachusetts USA; 12 Harvard-Massachusetts Institute of Technology Division of Health Sciences and Technology, Massachusetts Institute of Technology, Cambridge, Massachusetts USA; 13 Department of Materials Science and Engineering, University of California, Los Angeles, Los Angeles, California USA

**Keywords:** Biomedical engineering, Biomaterials, Materials chemistry, Techniques and instrumentation, Materials for devices

Correction to: *Nature Communications* 10.1038/s41467-025-59045-1, published online 22 April 2025

In the version of the article initially published, a thiophene ring in Fig. 1a was incorrectly drawn and has now been amended in the HTML and PDF versions of the article, as seen below:

**Fig. 1 | Original**

**Figure:**
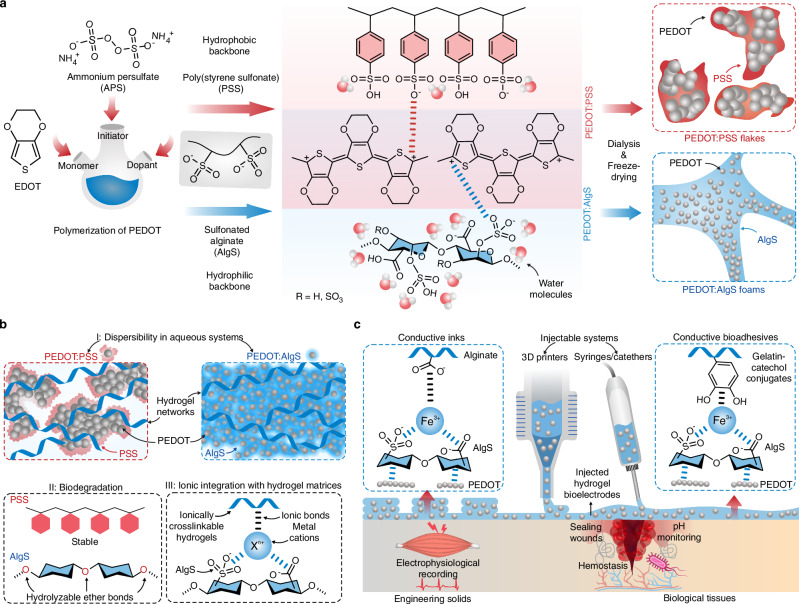

**Fig. 1 | Corrected**

**Figure:**