# Supplementary material for: Boosting hydrogel conductivity via water-dispersible conducting polymers for injectable bioelectronics
Source: Nat Commun. 2025 Apr 22;16:3755. doi: 10.1038/s41467-025-59045-1 (PMC12015517; doi:10.1038/s41467-025-59045-1)
Supplement: Supplementary file 1 — Supplementary Information [file 41467_2025_59045_MOESM1_ESM.pdf]

Supplementary Information for

Boosting hydrogel conductivity *via* water-dispersible conducting polymers for injectable bioelectronics

Hossein Montazerian, Elham Davoodi, Canran Wang, Farnaz Lorestani, Jiahong Li, Reihaneh Haghniaz, Rohan R. Sampath, Neda Mohaghegh, Safoora Khosravi, Fatemeh Zehtabi, Yichao Zhao, Negar Hosseinzadeh, Tianhan Liu, Tzung Hsiai, Alireza Hassani Najafabadi,* Robert Langer, Daniel G. Anderson, Paul S. Weiss,* Ali Khademhosseini,* and Wei Gao*

*Corresponding authors: weigao@caltech.edu, khademh@terasaki.org, psw@cnsi.ucla.edu, hassania@terasaki.org.

Table of Contents

Supplementary Figure 1 | Synthesis of sulfonated alginate as doping element in synthesis of PEDOT.

Supplementary Figure 2 | Characterization of sulfonated alginate.

Supplementary Figure 3 | Characterization of freeze-dried PEDOT:AlgS.

Supplementary Figure 4 | Conductivity of PEDOT dry films doped with sulfonated alginate.

Supplementary Figure 5 | Impedance spectroscopy of PEDOT dry films.

Supplementary Figure 6 | Scanning electron microscopy images of freeze-dried PEDOT products.

Supplementary Figure 7 | Characterization of PEDOT:AlgS re-dispersions in water after freeze-drying.

Supplementary Figure 8 | Ionic responsiveness of PEDOT:AlgS synthesized at various EDOT concentrations to Fe^{3+}.

Supplementary Figure 9 | Long-term in vivo degradation and immune response to intradermally injected PEDOT solutions.

Supplementary Figure 10 | Hydrolytic degradability of PEDOT additives.

Supplementary Figure 11 | Characterization of PEDOT films.

Supplementary Figure 12 | Comparison of the relative increase in matrix conductivity.

Supplementary Figure 13 | Circuit modeling of conductive alginate hydrogels incorporated with PEDOT additives.

Supplementary Figure 14 | Printability and SEM images of freeze-dried hydrogels of alginate with PEDOT additives.

Supplementary Figure 15 | In vitro toxicity study of $FeCl_3$ at varying concentrations using PrestoBlue assay of cell metabolic activity for human dermal fibroblast cells.

Supplementary Figure 16 | Immunomodulatory effects of PEDOT-based additives in alginate hydrogels.

Supplementary Figure 17 | Effect of PEDOT additives on thermal gel-sol transition of GelCA bioadhesive hydrogels.

Supplementary Figure 18 | Effect of PEDOT additives on tensile mechanical properties of GelCA bioadhesive hydrogels.

Supplementary Figure 19 | Electrical characterization of conductive GelCA-based composites comprising of PEDOT additives.
Supplementary Figure 20 | Assessment of wet stability of crosslinking and static adhesion of conductive bioadhesives to collagen sheets.
Supplementary Figure 21 | Hemostatic properties of conductive bioadhesives incorporated with PEDOT additives.
Supplementary Figure 22 | pH sensing capabilities of conductive bioadhesives.
Supplementary Figure 23 | In vitro biocompatibility study of conductive bioadhesives.
Supplementary Figure 24 | Wound healing and antibacterial effects of PEDOT additives in bioadhesive GelCA hydrogels.

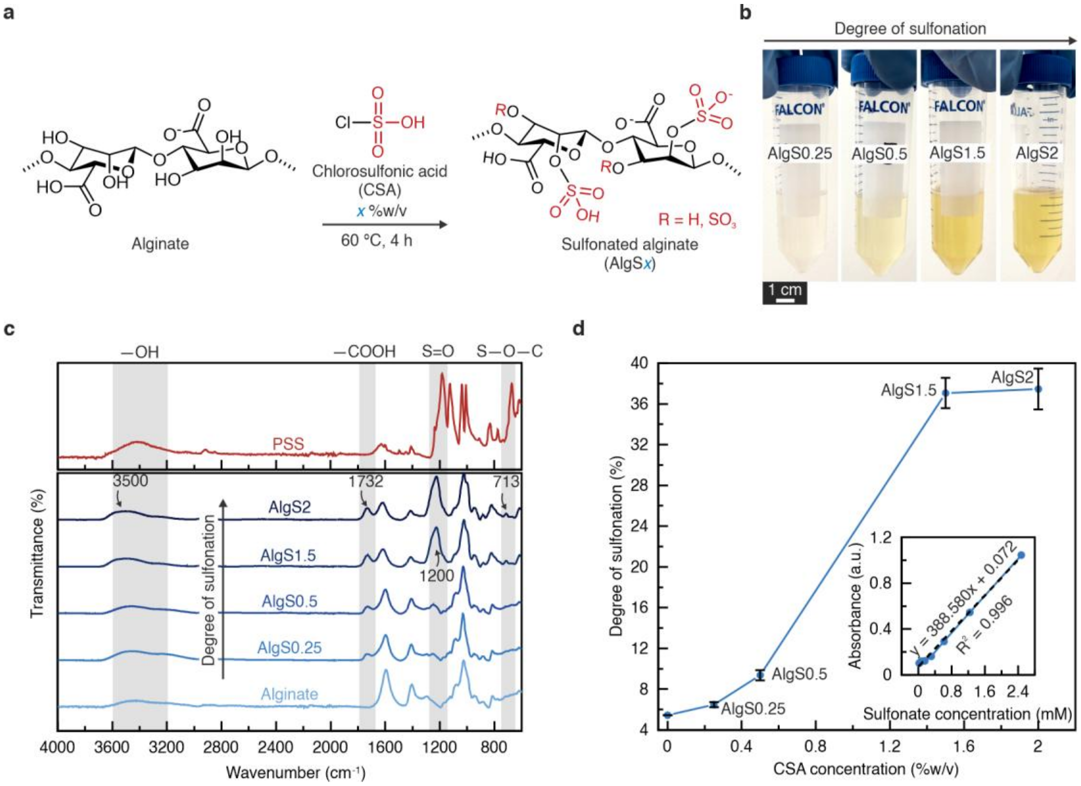

Supplementary Figure 1 | Synthesis of sulfonated alginate as doping element in synthesis of PEDOT. a, Reaction scheme for sulfonation of alginate to yield AlgS. **b**, Visual color changes of the reaction solution with varying degrees of sulfonation. Alginate solutions changed color from turbid and colorless to transparent and yellow as CSA concentration increased. **c**, Fourier transform infrared (FTIR) spectra of PSS and AlgS with various sulfonation degrees. The peak at 1200 cm^{-1} intensified with sulfonation, due to the S=O stretching vibration in SO_3 conjugates.[S1] The peak at 713 cm^{-1} corresponds to S—O—C vibration.[S1] The peak at 1732 cm^{-1} increasing with CSA is ascribed to the carbonyls in carboxylic acid groups.[S2] The wide shoulder over ~3400-3600 cm^{-1} range is related to stretching vibrations of hydroxyl (—OH) groups. **d**, Characterization of the degree of sulfonation with CSA concentration obtained from the colorimetric barium sulfate nephelometry tests. The inset shows the calibration curves used for the calculation of the degree of sulfonation. Data represents mean ± standard deviation (n=3 independent samples).

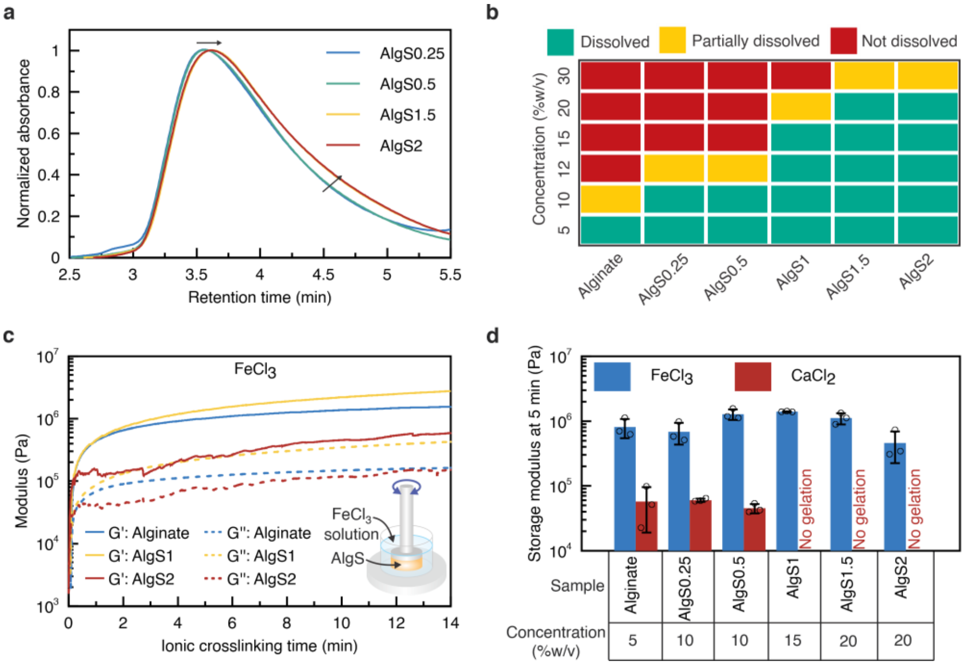

Supplementary Figure 2 | Characterization of sulfonated alginate. **a**, Size exclusion chromatography studies for the characterization of molecular weight distribution of AlgS with varying degrees of sulfonation. Negligible peak shift to the right suggests minimal chain degradation during the sulfonation reaction likely due to acidic hydrolysis and heating. **b**, Effect of sulfonation degree on water solubility of alginate. **c**, Effect of sulfonation degree on ionic crosslinking of AlgS solutions at their solubility limits in response to 100 mM $FeCl_3$ solutions. **d**, Storage modulus of AlgS (at their solubility concentrations) after 5 min exposure to 100 mM $FeCl_3$ and $CaCl_2$ solutions. The data represent mean ± standard deviation (n=3 independent samples).

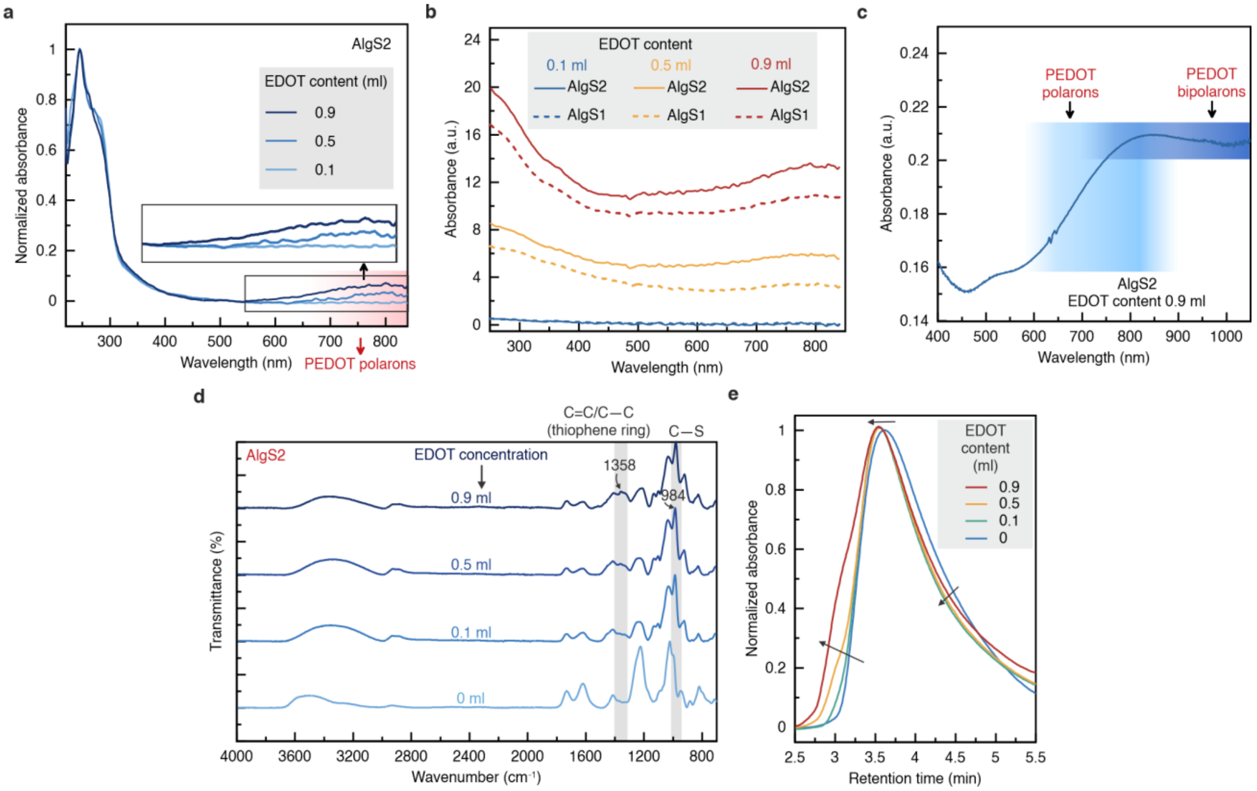

Supplementary Figure 3 | Characterization of freeze-dried PEDOT:AlgS. **a**, UV-vis absorption spectra of PEDOT:AlgS synthesized at different EDOT contents dispersed in water. The shoulder peak at wavelengths > 700 nm suggests that PEDOT stayed in doped state after dialysis and freeze-drying processes (PEDOT polymerized for 1 d). **b**, Effects of EDOT content and alginate sulfonation degree on UV-vis spectra of PEDOT:AlgS. The magnitude of the absorbance was larger for higher sulfonation and EDOT contents (PEDOT polymerized for 1 d). **c**, UV-vis spectra of PEDOT:AlgS at the near-infrared wavelength ranges. **d**, FTIR spectra of PEDOT:AlgS synthesized at different EDOT contents. **e**, Results of size exclusion chromatography characterizing molecular weight distribution of PEDOT:AlgS at varying EDOT contents (PEDOT polymerized for 1 d).

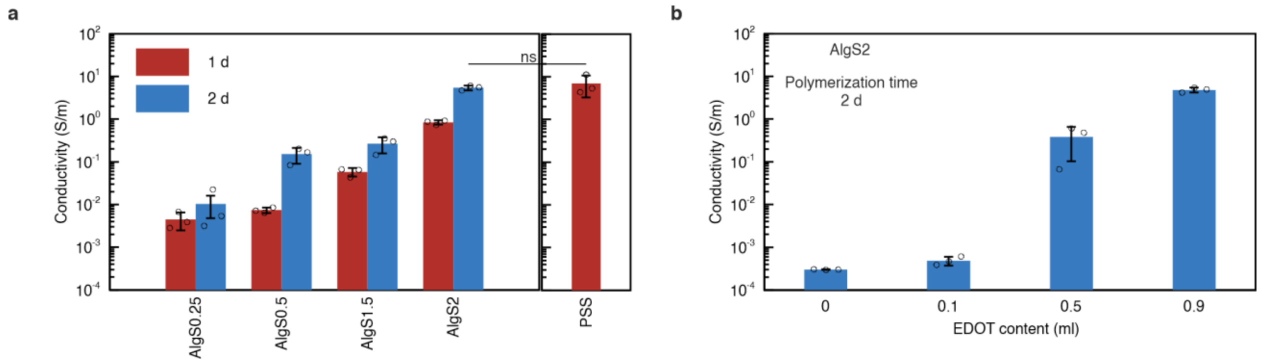

Supplementary Figure 4 | Conductivity of PEDOT dry films doped with sulfonated alginate. The films were dried from the reaction solutions before dialysis and freeze-drying steps. **a**, Effect of alginate sulfonation degree and EDOT polymerization time on the conductivity of PEDOT:AlgS and comparisons with PEDOT:PSS. **b**, Effect of EDOT monomer concentration on the conductivity of PEDOT:AlgS2 after 2 days of EDOT polymerization reaction. The data represent mean ± standard deviation for three independent samples.

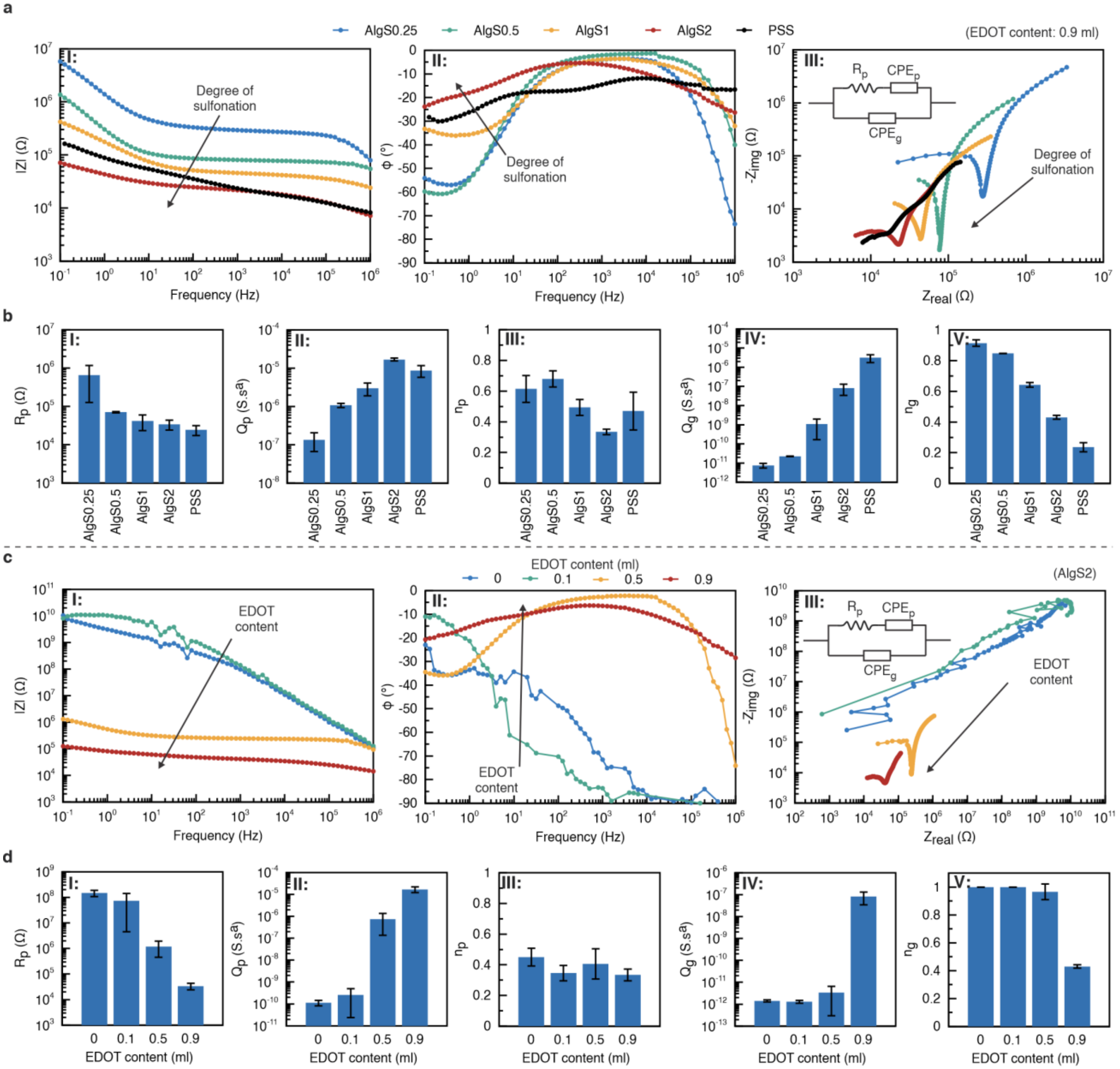

Supplementary Figure 5 | Impedance spectroscopy of PEDOT dry films. Polymerization of PEDOT:AlgS and PEDOT:PSS was performed in 2 and 1 days, respectively, and the films were air-dried from the reaction solutions before dialysis and freeze-drying steps. **a**, I: Impedance magnitude plots, II: phase diagrams, and III: Nyquist plots for dry PEDOT:PSS and PEDOT:AlgS with different sulfonation degrees synthesized at 0.9% w/v EDOT content. Here, the constant phase element (CPE_g) combines the effects of geometric CPE and insulating defects such as porosity and cracks. The resistance R_p in series with CPE_p together represent the interconnected dual phase structure of PEDOT and its dopant. The proposed circuit fit well with experimental

data ($1.8\times10^{-4} < \chi^2 < 2.1\times10^{-2}$). **b**, The results of fitting the impedance data to the equivalent circuit are shown in the inset of a. III: The capacitive constants and exponential values for both CPEs increased with sulfonation degree, which implies closer behavior to ideal resistance due to the greater PEDOT doping *via* sulfonate groups. **c**, I: Impedance magnitude plots, II: phase diagrams, and III: Nyquist plots for dry PEDOT:AlgS2 synthesized at different EDOT content. **d**, Effects of EDOT content on fitting parameters for the equivalent circuit model shown in the inset of cIII. Similar to b, the capacitive constants and exponential values for both CPEs increased with EDOT content due to the promoted PEDOT interconnectivity. R_p is the resistance of its corresponding resistor, Q_p and Q_g, as well as n_p and n_g are constants and exponential values of constant phase elements CPE_p and CPE_g, respectively. The data represent mean ± standard deviation (n=3 independent samples).

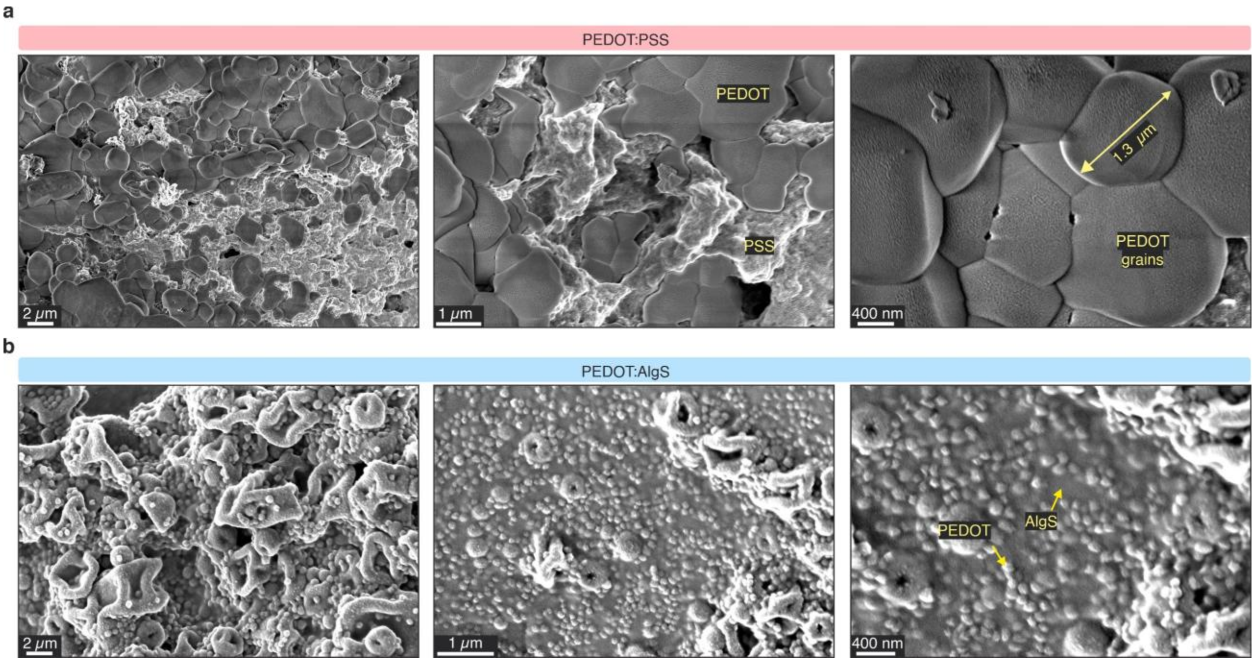

Supplementary Figure 6 | SEM images of freeze-dried PEDOT products. a, PEDOT:PSS, and **b**, PEDOT:AlgS (n = 3 samples with similar results).

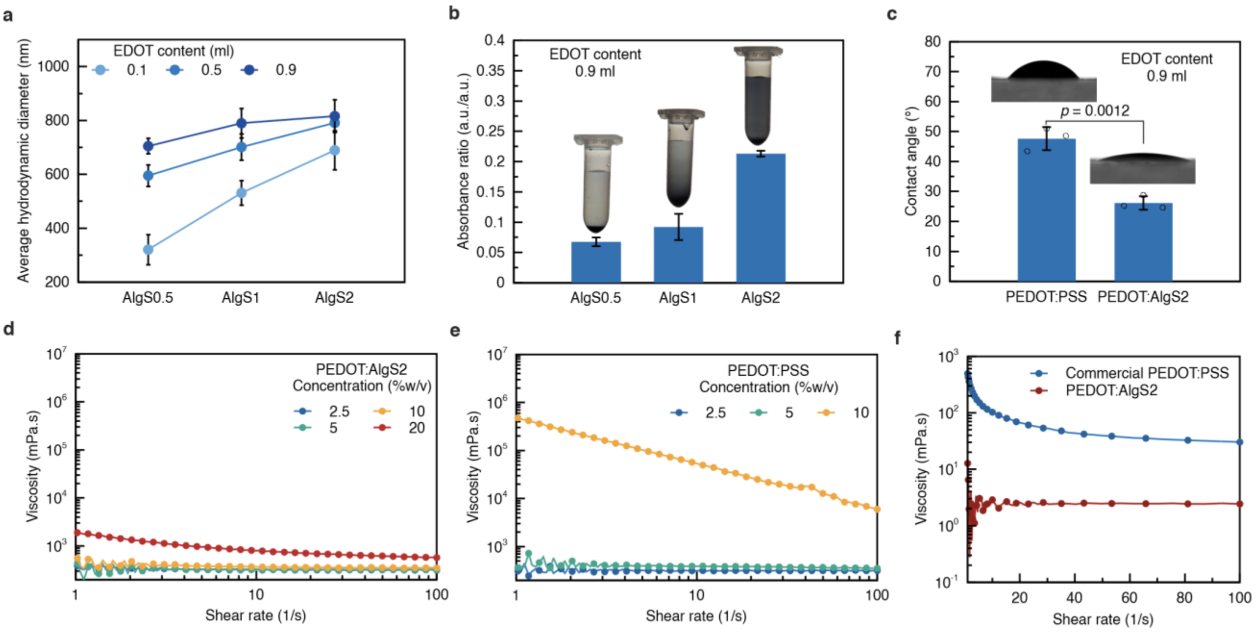

Supplementary Figure 7 | Characterization of PEDOT:AlgS redispersions in water after freeze-drying. a, Effects of sulfonation degree and EDOT monomer on the average hydrodynamic size results from DLS tests (PEDOT polymerized for 1 d). **b**, Colloidal stability of PEDOT:AlgS measured in terms of the ratio of supernatant absorbance (after the 0.5% w/v solutions were left at rest for three months) to the absorbance of homogeneous solution of PEDOT:PSS and PEDOT:AlgS2 in water (PEDOT polymerized for 1 d). **c**, Water contact angle of PEDOT:PSS and PEDOT:AlgS coatings air-dried on glass substrates. Student's t-test was performed to determine statistical significance. **d**,**e**, Viscosity-shear rate characteristics of PEDOT:AlgS and PEDOT:PSS solutions, respectively, in water at different concentrations. **f**, Comparison of viscosity-shear rate curves of commercial PEDOT:PSS and synthesized PEDOT:AlgS prepared at a similar PEDOT to dopant ratio (1:2.5) and dispersed at the same concentration in water (1.3 wt.%). The data represent mean ± standard deviation for three independent samples. The standard deviation for **b** was obtained *via* propagation of uncertainty.

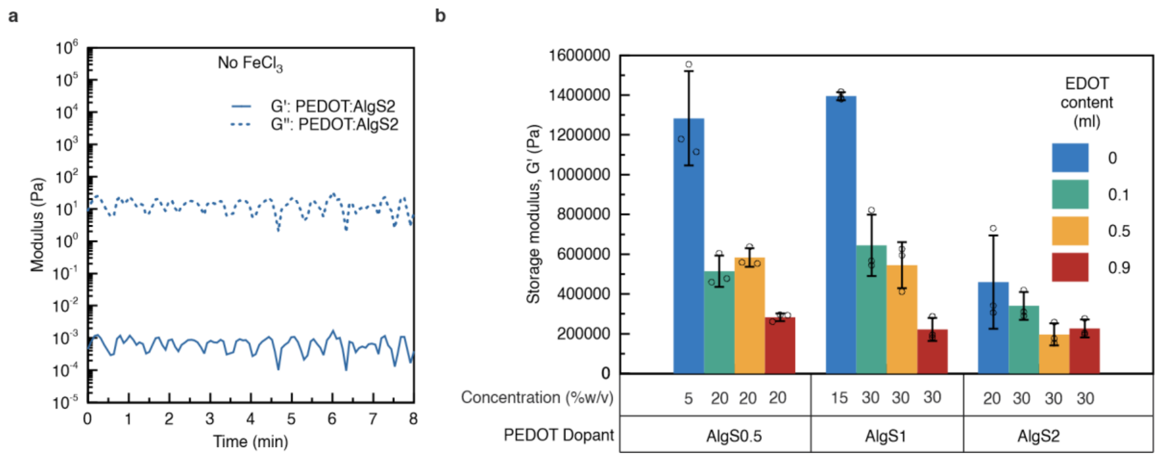

Supplementary Figure 8 | Ionic responses of PEDOT:AlgS synthesized at various EDOT contents to Fe^{3+}. a, Rheological variations of PEDOT:AlgS2 in the absence of Fe^{3+} ions. **b**, Results represent response to Fe^{3+} diffusion in terms of storage modulus (G′) after 5 min post-treatment with 100 mM $FeCl_3$ solutions. The PEDOT:AlgS solutions were prepared at their dispersibility concentration limit (PEDOT polymerized for 1 d). The data represent mean ± standard deviation (n=3 independent samples).

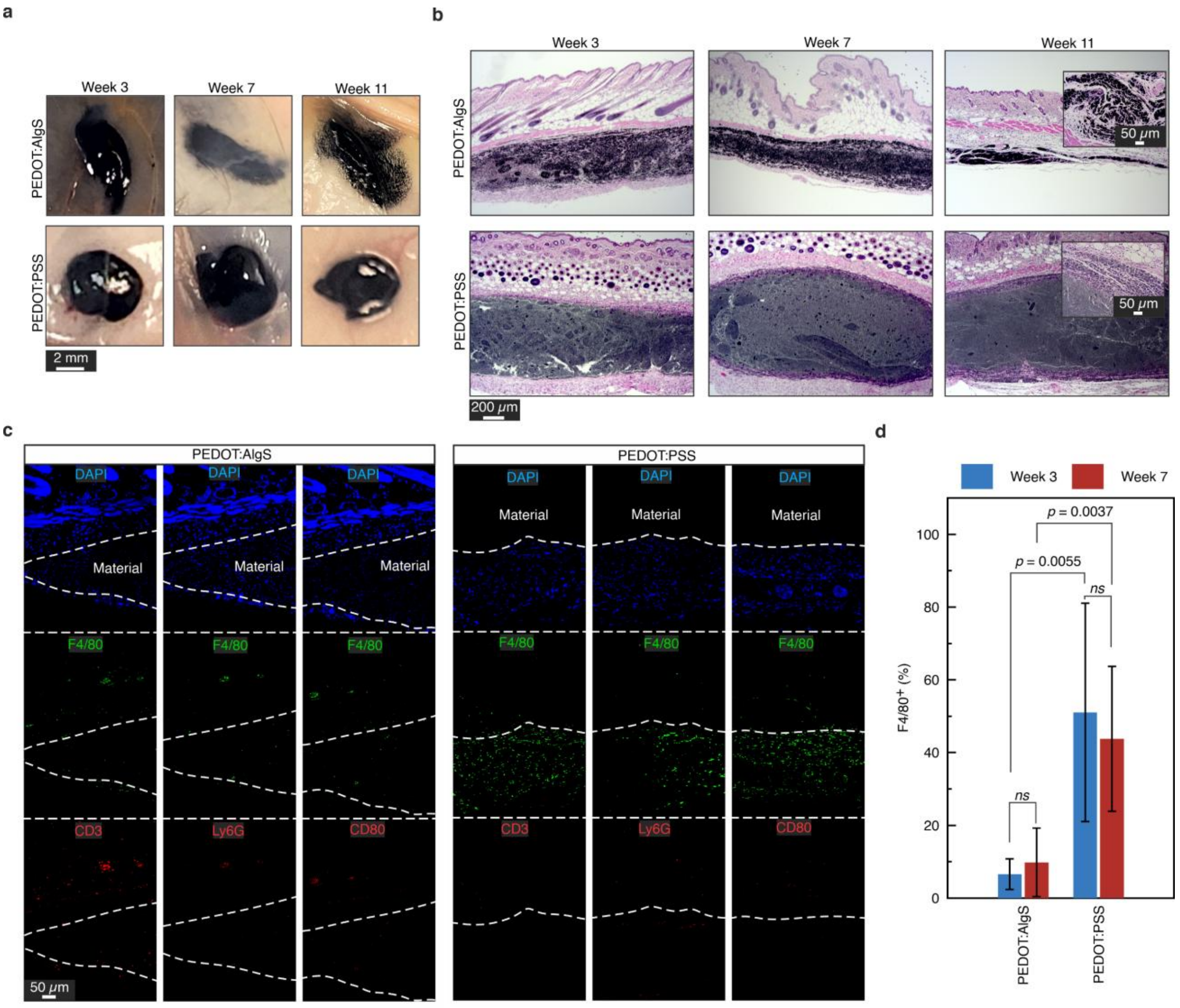

Supplementary Figure 9 | Long-term *in vivo* degradation and immune response to intradermally injected 5 w/v% PEDOT solutions. a, Images of injected PEDOT at different timepoints. **b**, H&E staining images of PEDOT:PSS and PEDOT:AlgS (n = 3 samples with similar results). **c**, Immunostaining results using DAPI (blue), F4/80 (green), CD3, Ly6G, and CD80 (red) markers for week 3 after implantation. **d**, The ratio of F4/80$^+$ cells to the total cells under the muscle layer. The data represent mean ± standard deviation (n=3 independent samples) and the statistical significance was analyzed *via* two-way ANOVA (*ns*, p>0.05).

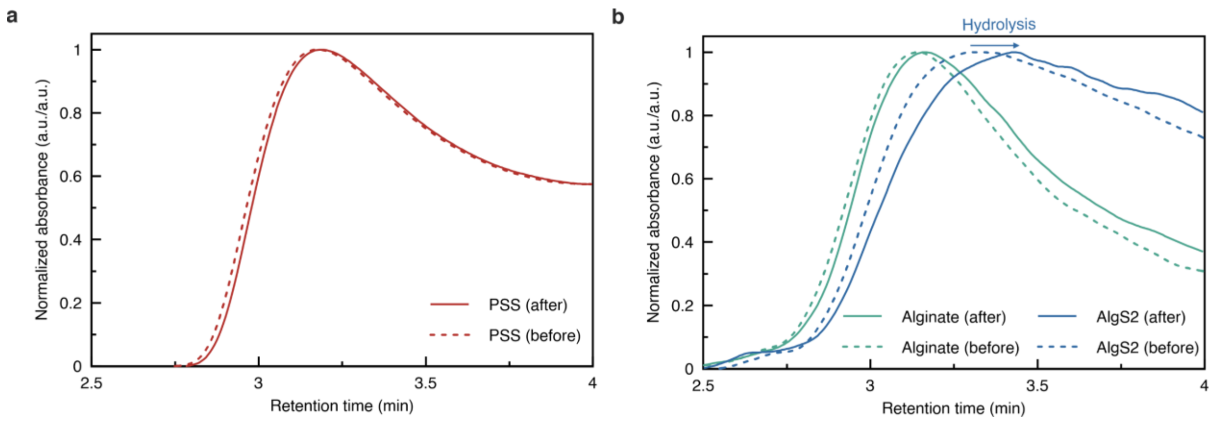

Supplementary Figure 10 | Hydrolytic degradability of dopants. The SEC curves obtained for **a**, PSS, **b**, alginate and its sulfonated products (AlgS). The curves represent molecular weight characteristics resulting from SEC tests before and after treatment in alkaline conditions.

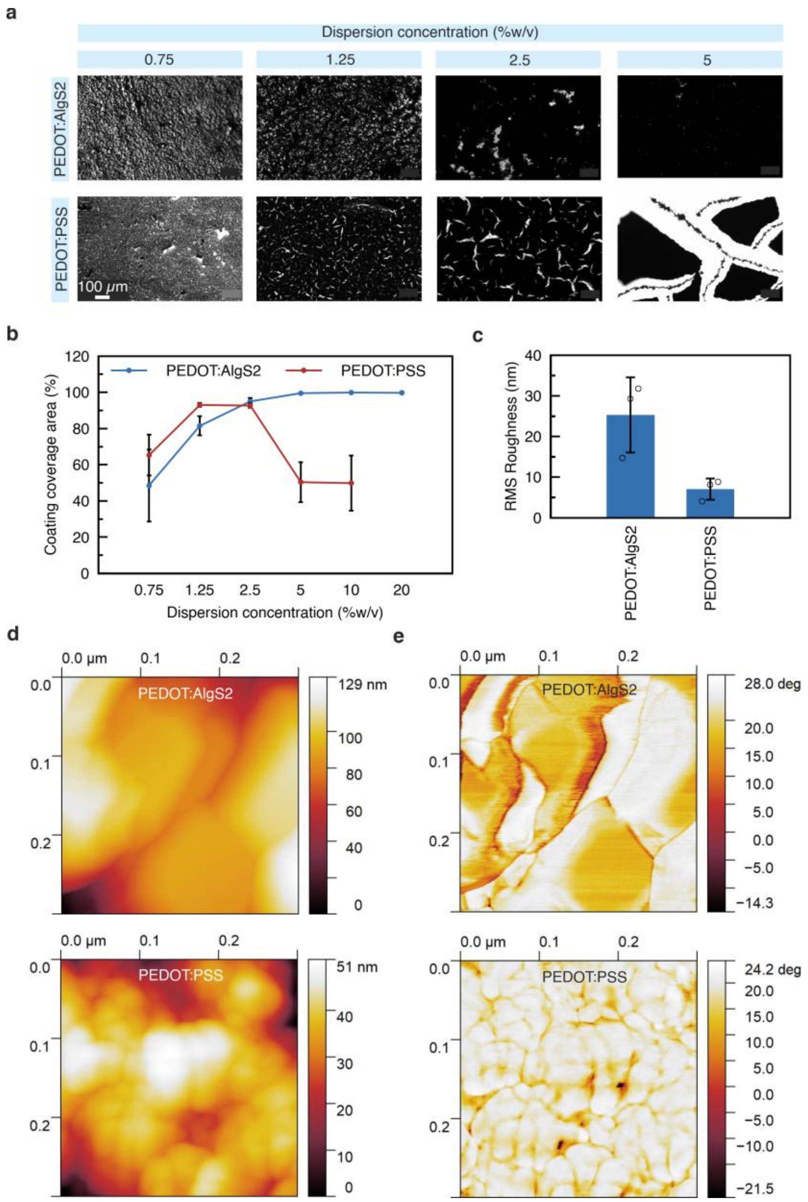

Supplementary Figure 11 | Characterization of PEDOT films. a, Microscope images of the coatings from various concentrations of PEDOT:PSS and PEDOT:AlgS2. **b**, Coating coverage ratio for PEDOT:PSS and PEDOT:AlgS coatings on glass substrates. **c**, Comparing the root mean square (RMS) roughness of PEDOT:PSS and PEDOT:AlgS coatings obtained by atomic force microscopy (AFM). The data in **b** and **c** represent mean ± standard deviation (n=3 independent samples). **d**,**e**, The z-plots and phase plots, respectively, for PEDOT:AlgS2 and PEDOT:PSS samples from AFM imaging of coatings obtained from 2.5% w/v PEDOT solutions (n = 3 samples with similar results).

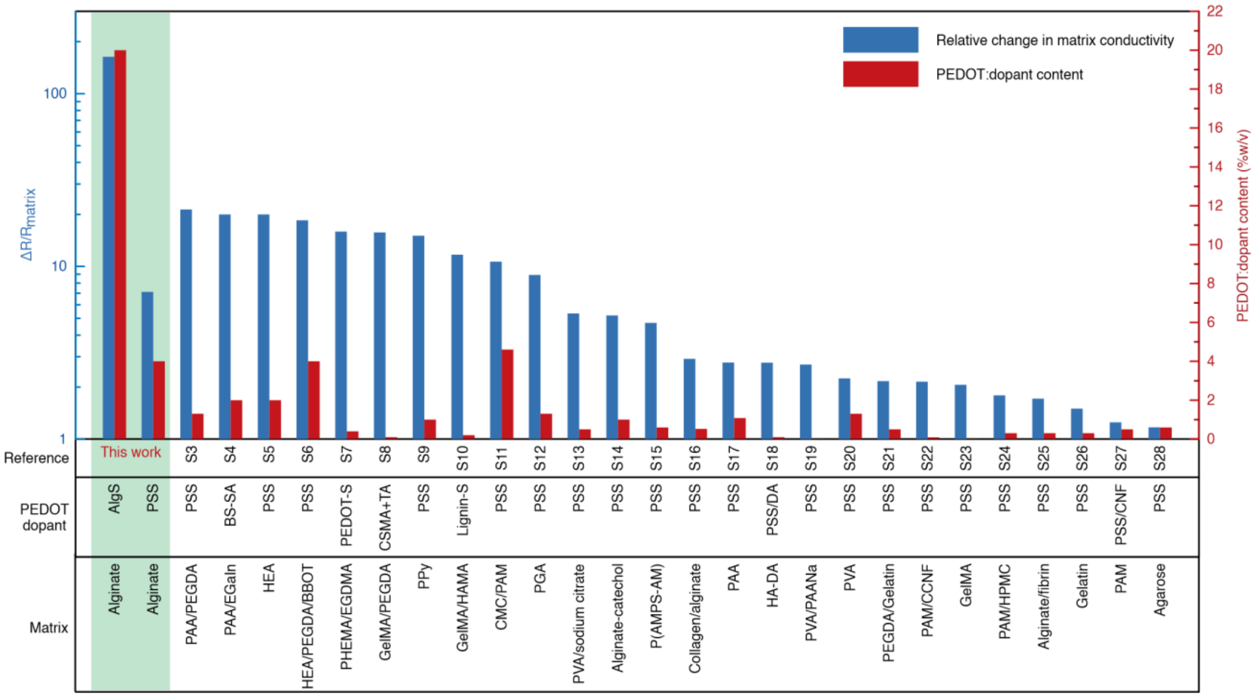

Supplementary Figure 12 | Comparison of the relative increase in matrix conductivity. High conductivity enabled by large dispersibility of PEDOT:AlgS in aqueous hydrogel systems compared with those PEDOT-based hydrogel composites reported in Refs S3–S28. PAA, poly(acrylic acid); PEGDA, polyethylene glycol diacrylate; EGaln, gallium-Indium eutectic; HEA, 2-hydroxyethyl acrylate; BBOT, 2,5-bis(5-tert-butyl-2-benzoxazolyl thiophene; PHEMA, poly(2-hydroxyethyl methacrylate); GelMA, gelatin methacryloyl; PPy, polypyrrole; HAMA, hyaluronic acid methacrylate; CMC, carboxymethyl cellulose; PGA, poly(glycolic acid); PVA, γ-polyglutamic acid; P(AMPS-AM), poly(2-acrylamido-2-methylpropanesulfonic acid)/poly(acrylamide); HA-DA, dopamine-modified hyaluronic acid; PAANa, poly(acrylic acid) sodium salt; CCNF, carboxylic cellulose nanofibers; PAM, poly(acrylamide); HPMC, hydroxypropyl methyl cellulose.

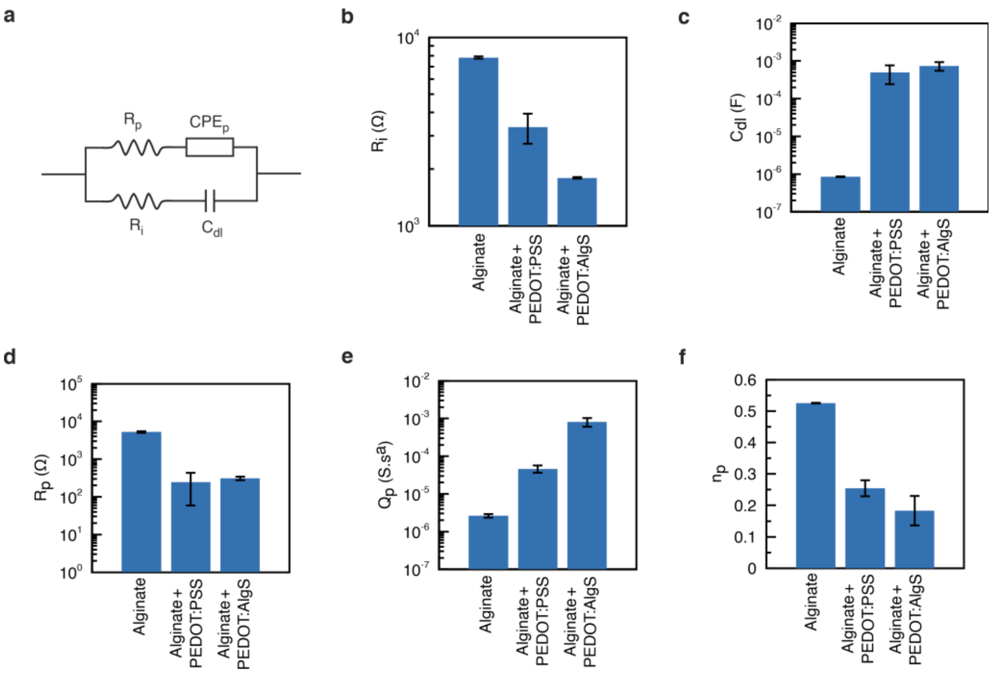

Supplementary Figure 13 | Circuit modeling of conductive alginate hydrogels incorporated with PEDOT additives. **a**, The equivalent circuit for fitting the impedance data. Assuming negligible charge transfer at the electrodes **b**, a resistor R_i (representative of ionic conduction) in series with a **c**, capacitor C_{dl} (double-layer capacitance) was modeled. PEDOT:AlgS led to ~45% lower ionic resistance than PEDOT:PSS. These elements were modeled in parallel with **d**, a resistor R_p and **e**, constant phase element CPE_p (accounting for the insulating components inhibiting PEDOT-PEDOT direct contacts (such as alginate and PSS). The constants obtained from circuit fitting, *i.e.*, R_i, C_{dl}, R_p, Q_p, and n_p. R_i and R_p are resistances of their corresponding resistors in a, C_{dl} is the capacitive constant of capacitor C_{dl}, Q_p, and n_p are the constant and exponential values of the capacitive phase element (CEP_p). The data represent mean ± standard deviation (n=3 independent samples).

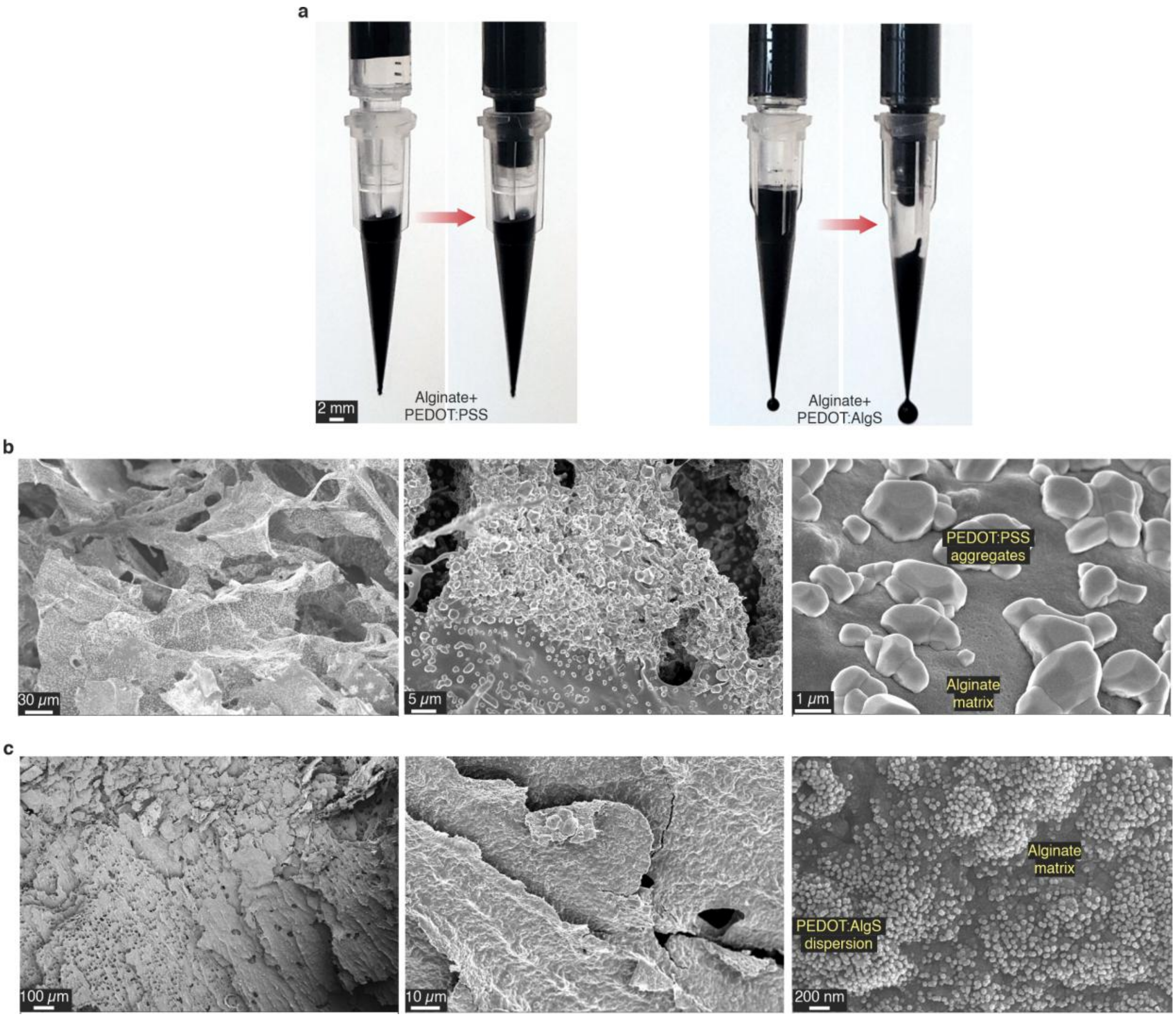

Supplementary Figure 14 | Printability and SEM images of alginate-PEDOT composites. a, Clogging observed in the printing nozzle for alginate+PEDOT:PSS inks, compared to the smooth flow of alginate+PEDOT:AlgS through the needle gauge. Air trapped in the syringe demonstrates the air pressure effect on the inks. **b,c,** SEM images of alginate-PEDOT hydrogels containing **b**, 4% w/v PEDOT:PSS and **c**, 20% w/v PEDOT:AlgS (n = 3 samples with similar results).

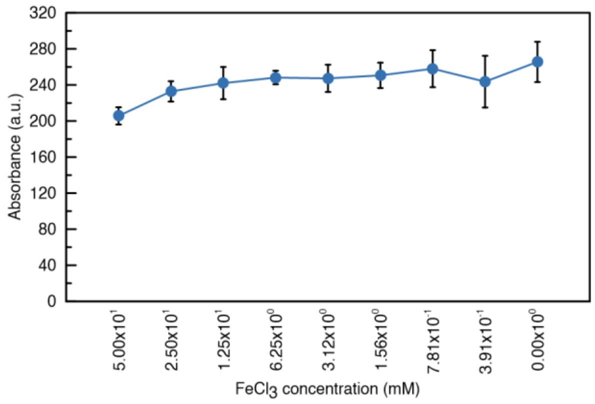

Supplementary Figure 15 | *In vitro* toxicity study of $FeCl_3$ at varying concentrations using PrestoBlue assay of cell metabolic activity for human dermal fibroblast cells. The data represent mean ± standard deviation (n=3 independent samples).

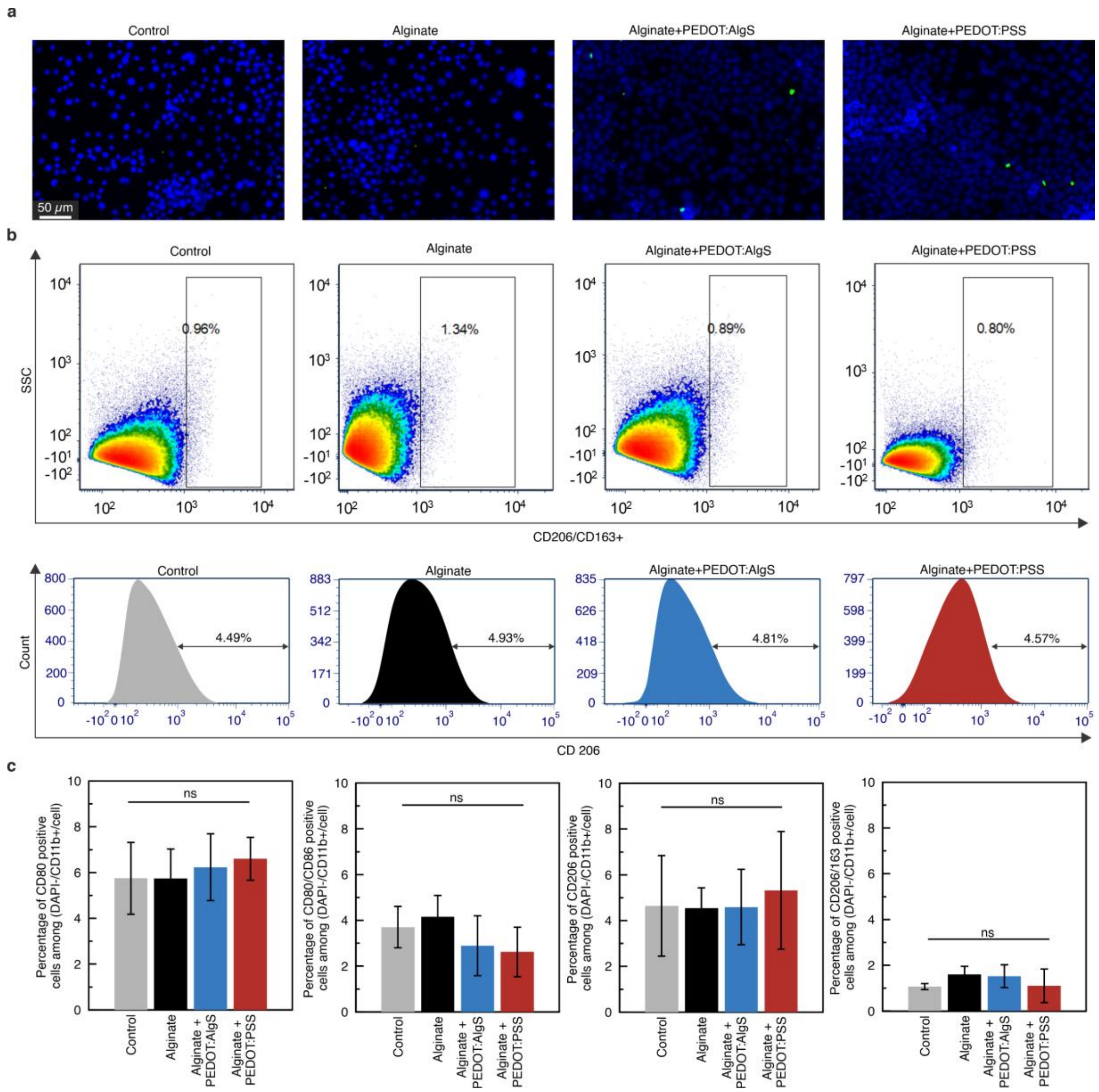

Supplementary Figure 16 | Immunomodulatory effects of PEDOT-based additives in alginate hydrogels. a, Immunofluorescent staining on bone-marrow-derived macrophages (BMDM) using CD80 markers and nuclear staining. Widefield fluorescent images were captured after 24 h, with color-coding: green to CD80 and blue to DAPI. **b**, The flow cytometry results of BMDM cells co-cultured with hydrogels for 24 h. **c**, Flow cytometry quantification of pro-inflammatory M1 and anti-inflammatory M2 markers. Blank samples were used as controls. The data represent mean ± standard deviation (n=3 independent samples) obtained *via* propagation of uncertainty (*ns*, p>0.05).

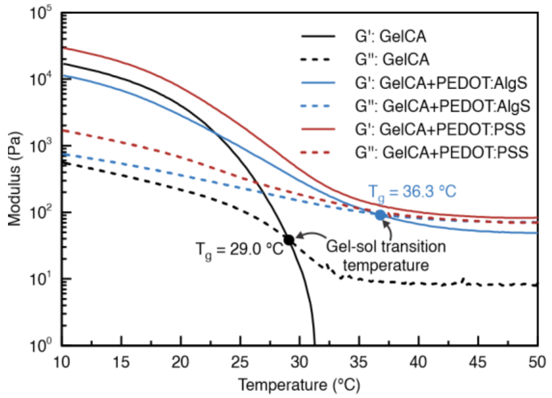

Supplementary Figure 17 | Effect of PEDOT additives on thermal gel-sol transition of GelCA bioadhesive hydrogels. The gel-sol transition point is designated as the crossover of storage (G′) and loss (G″) modulus.

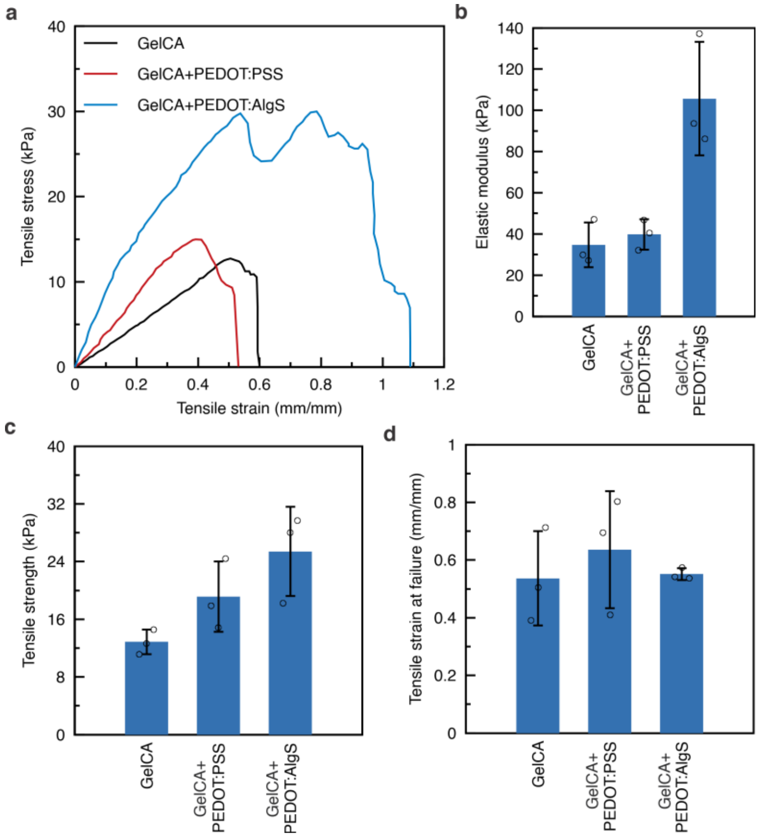

Supplementary Figure 18 | Effect of PEDOT additives on thermal gelation of GelCA bioadhesive hydrogels. a, Stress-strain curves and the resulting **b**, elastic modulus, **c**, tensile strength, and **d**, tensile strain at failure (stretchability) of the ionically crosslinked hydrogels. The data represent mean ± standard deviation (n=3 independent samples).

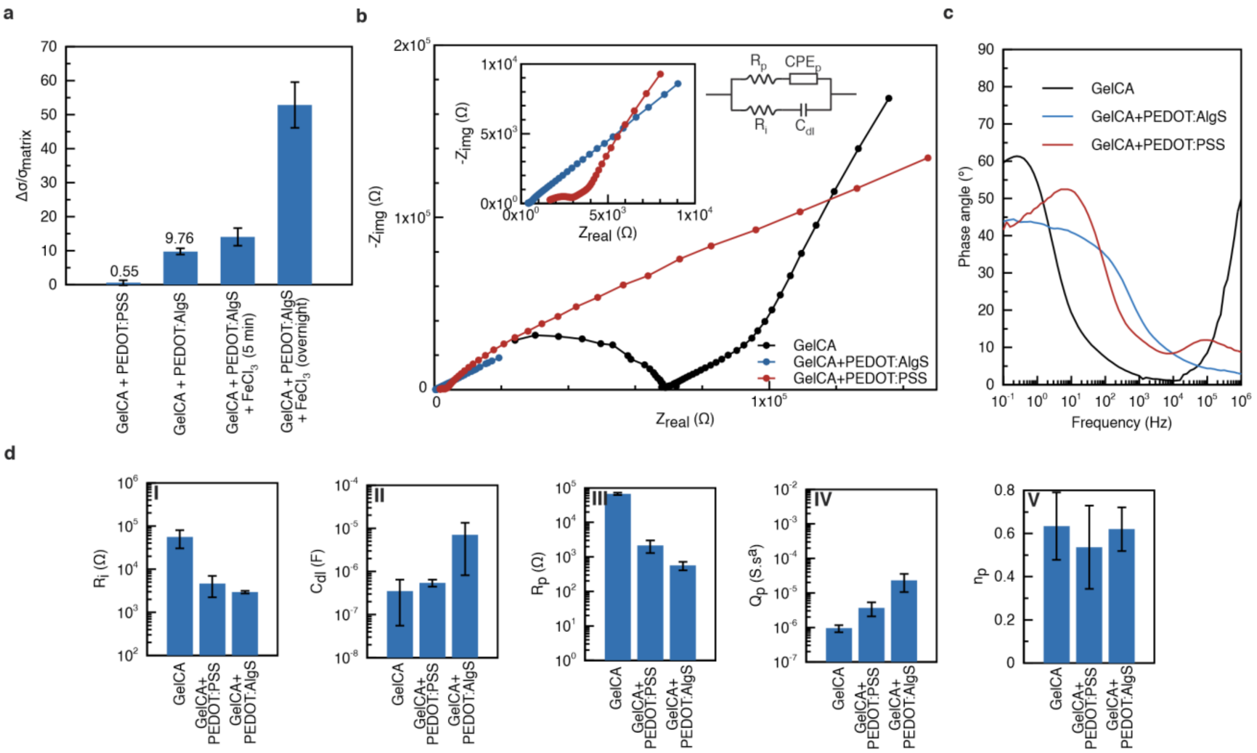

Supplementary Figure 19 | Electrical characterization of conductive GelCA-based composites comprising of PEDOT additives. a, The relative increase in conductivity of 12% w/v GelCA matrices (2.9×10^{-4} S m^{-1}) with PEDOT:AlgS and PEDOT:PSS at their dispersibility limit before and after diffusive crosslinking in 25 mM $FeCl_3$. Error bars are standard deviations obtained by propagation of uncertainty. **b**, **c**, Nyquist plots and phase Bode plots, respectively, obtained from impedance spectroscopy for GelCA solutions and its composites with synthesized PEDOT additives at their dispersibility limits. Z_{img} and Z_{real} are imaginary and real parts of impedance. **d**, The results of fitting the impedance data to the equivalent circuit model shown in the inset of **b**. R_i and R_p are resistances of their corresponding resistors in **b**, C_{dl} is the capacitive constant of capacitor C_{dl}, Q_p, and n_p are the constant and exponential values of the capacitive phase element (CEP_p). The data represent mean ± standard deviation (n=3 independent samples).

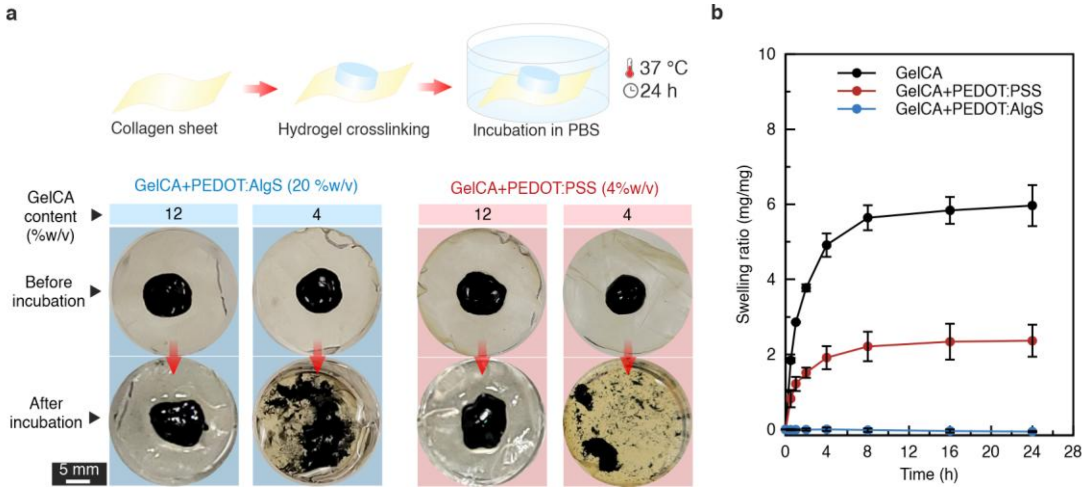

Supplementary Figure 20 | Assessment of wet stability of crosslinking and static adhesion of conductive bioadhesives to collagen sheets. a, Static adhesion of bioadhesive GelCA loaded with PEDOT:PSS and PEDOT:AlgS at their dispersibility limits after incubation for 24 h at 37 °C. **b**, Swelling kinetic of crosslinked hydrogels incubated in water. The data represent mean ± standard deviation (n=3 independent samples).

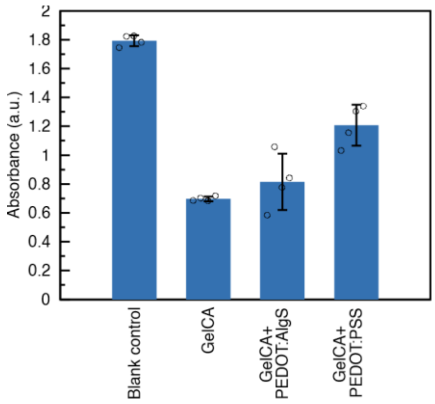

Supplementary Figure 21 | Hemostatic properties of conductive bioadhesives incorporated with PEDOT additives. The data represent mean ± standard deviation (n=4 independent samples).

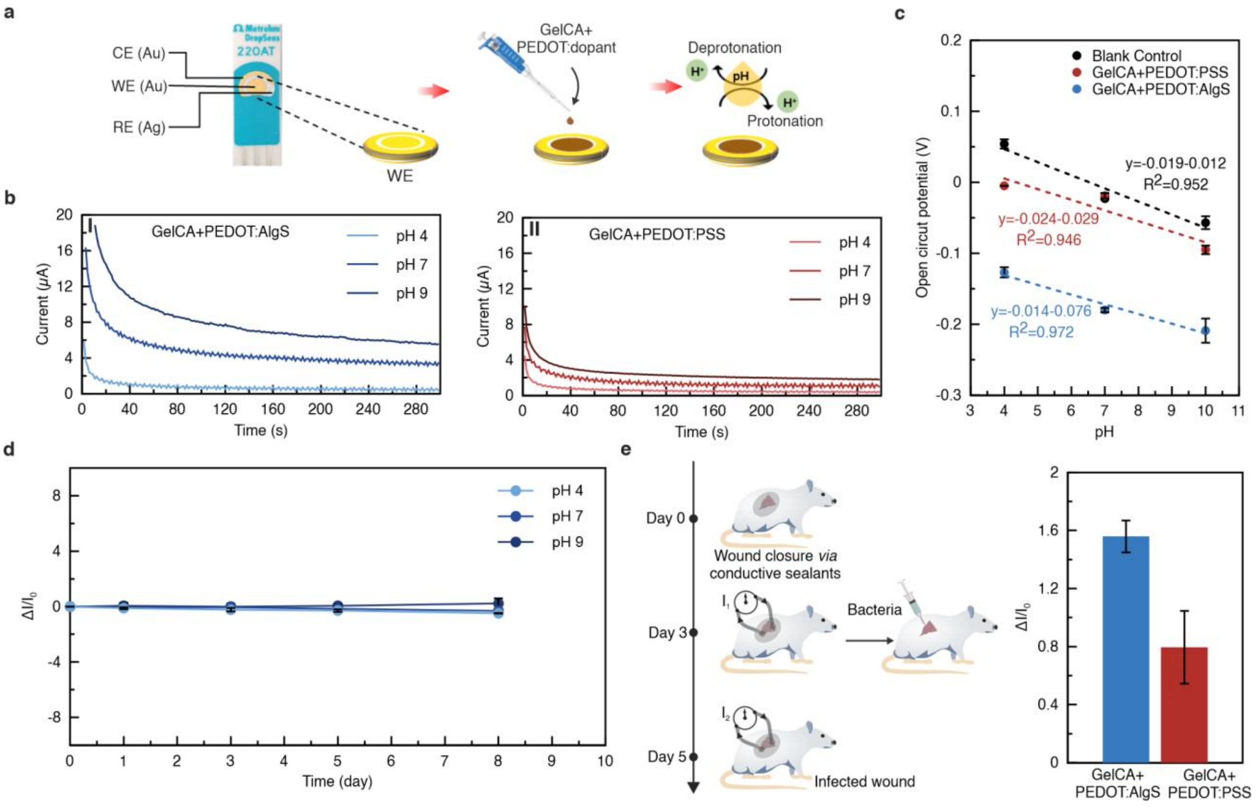

Supplementary Figure 22 | pH sensing capabilities of conductive bioadhesives. a,**b**, Real-time data of chronoamperometry tests showing the dependence of conductivity to different pH values for GelCA (12% w/v) + PEDOT:PSS (4% w/v) and + PEDOT:AlgS (20% w/v), respectively. The tests were conducted in buffer solutions with varying pH levels (pH 4, 7, and 9) while applying a potential of 0.65 V. **c**, Open circuit potential results comparing pH sensitivity of bioadhesives. **d,** Current stability of the crosslinked GelCA+PEDOT:AlgS hydrogels at different pH levels. **e,** *In vivo* sensing of wound infection after 3 days post wound closure. CE, counter electrode; WE, working electrode; RE, reference electrode. The data represent mean ± standard deviation (n=3 independent samples).

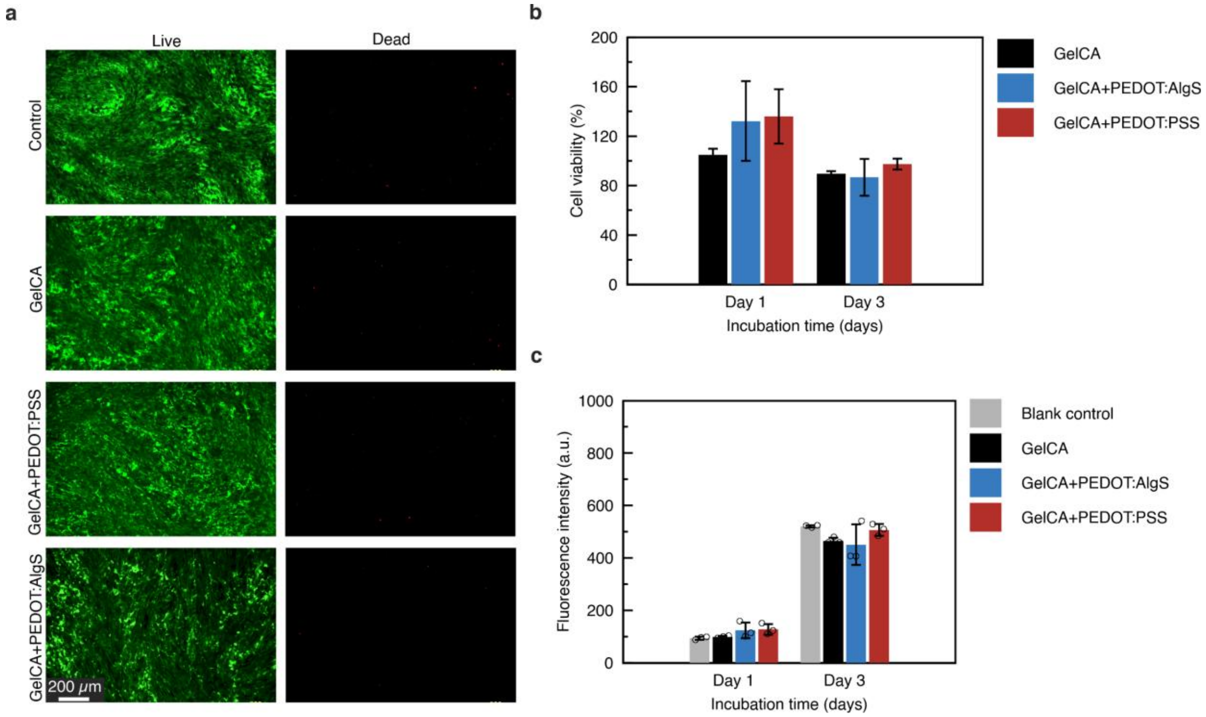

Supplementary Figure 23 | *In vitro* biocompatibility study of conductive bioadhesives. a, Live/Dead images of cells co-cultured with human dermal fibroblast cells. **b**, Cell viability results calculated based on the Live/Dead images. **c**, Results of metabolic PrestoBlue assays. Hydrogel formulations involve 12% w/v GelCA and 4% w/v PEDOT:PSS or 20% w/v PEDOT:AlgS. The data represent mean ± standard deviation (n=3 independent samples).

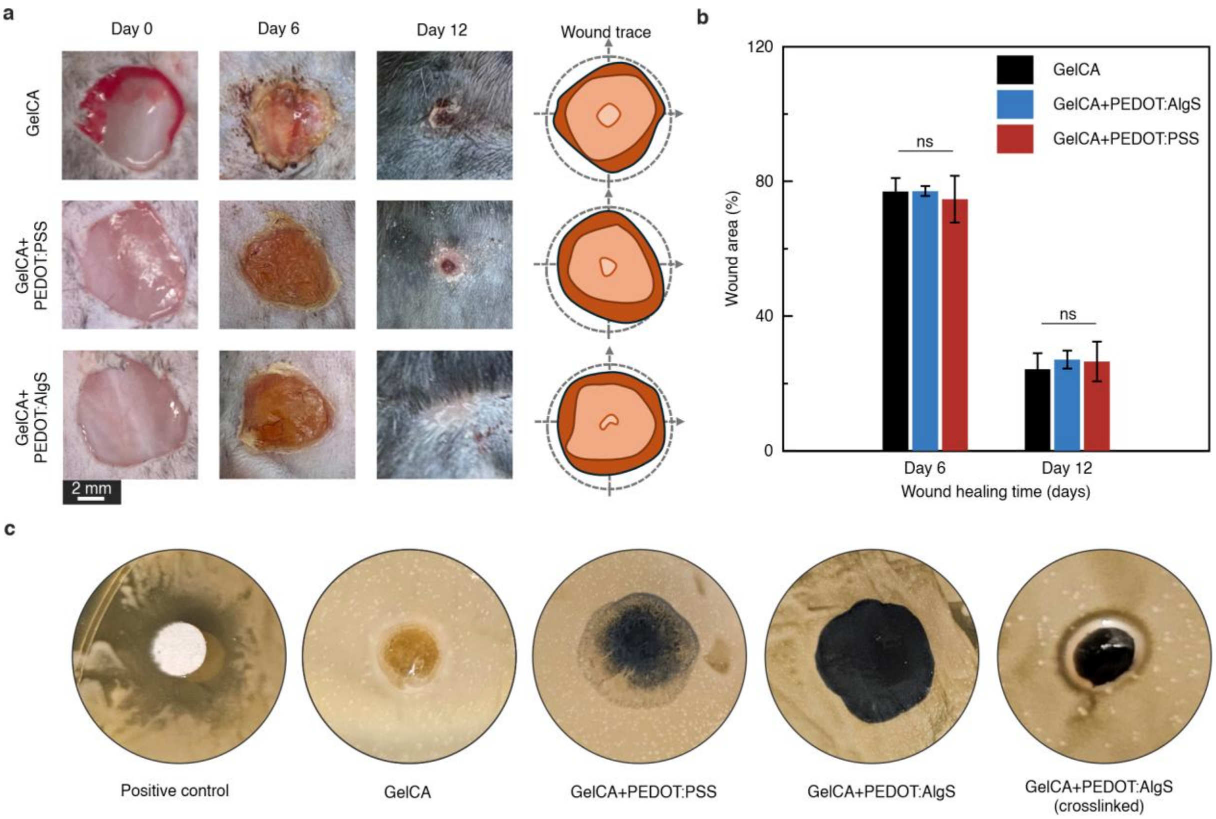

Supplementary Figure 24 | Wound healing and antibacterial effects of PEDOT additives in bioadhesive GelCA hydrogels. **a**, *In vivo* healing of skin wounds after 6 and 12 days using electroconductive bioadhesive dressings. **b**, Relative decrease in wound area calculated based on the wound traces. **c**, Zone of inhibition antibacterial tests against *E. coli* bacterial strain for GelCA bioadhesives supplemented with PEDOT:PSS and PEDOT:AlgS before and after crosslinking. The data in **b** represent mean ± standard deviation (n=3), *ns*, $p>0.05$.

SUPPORTING REFERENCES

S1. Mohammadi S, Ramakrishna S, Laurent S, Shokrgozar MA, Semnani D, Sadeghi D, *et al.* Fabrication of nanofibrous PVA/alginate-sulfate substrates for growth factor delivery. *J. Biomed. Mater. Res. Part A* **107**, 403-413 (2019).

S2. Ronghua H, Yumin D, Jianhong Y. Preparation and *in vitro* anticoagulant activities of alginate sulfate and its quaterized derivatives. *Carbohydr. Polym.* **52**, 19-24 (2003).

S3. Zhu H, Hu X, Liu B, Chen Z, Qu S. 3D printing of conductive hydrogel–elastomer hybrids for stretchable electronics. *ACS Appl. Mater. Interfaces* **13**, 59243-59251 (2021).

S4. Wang M, Feng X, Wang X, Hu S, Zhang C, Qi H. Facile gelation of a fully polymeric conductive hydrogel activated by liquid metal nanoparticles. *J. Mater. Chem. A* **9**, 24539-24547 (2021).

S5. Lopez-Larrea N, Gallastegui A, Lezama L, Criado-Gonzalez M, Casado N, Mecerreyes D. Fast visible-light 3D printing of conductive PEDOT:PSS hydrogels. *Macromol. Rapid Commun.* **45**, 2300229 (2023).

S6. Cheng X-Y, Peng S-Q, Wu L-X, Sun Q-f. 3D-printed stretchable sensor based on double network PHI/PEDOT:PSS hydrogel annealed with cosolvent of H_2O and DMSO. *Chem. Eng. J.* **470**, 144058 (2023).

S7. Keate RL, Tropp J, Collins CP, Ware HOT, Petty II AJ, Ameer GA, *et al.* 3D-printed electroactive hydrogel architectures with sub-100 µm resolution promote myoblast viability. *Macromol. Biosci.* **22**, 2200103 (2022).

S8. Song S, Li Y, Huang J, Cheng S, Zhang Z. Inhibited astrocytic differentiation in neural stem cell-laden 3D bioprinted conductive composite hydrogel scaffolds for repair of spinal cord injury. *Biomater. Adv.* **148**, 213385 (2023).

S9. Yang T, Xu C, Liu C, Ye Y, Sun Z, Wang B, *et al.* Conductive polymer hydrogels crosslinked by electrostatic interaction with PEDOT:PSS dopant for bioelectronics application. *Chem. Eng. J.* **429**, 132430 (2022).

S10. Gao C, Li Y, Liu X, Huang J, Zhang Z. 3D bioprinted conductive spinal cord biomimetic scaffolds for promoting neuronal differentiation of neural stem cells and repairing of spinal cord injury. *Chem. Eng. J.* **451**, 138788 (2023).

S11. Chen C, Li Y, Qian C, Liu X, Yang Y, Han L, *et al.* Carboxymethyl cellulose assisted

PEDOT in polyacrylamide hydrogel for high performance supercapacitors and self-powered sensing system. *Eur. Polym. J.* **179**, 111563 (2022).

S12. Zhang C, Wang M, Jiang C, Zhu P, Sun B, Gao Q, *et al.* Highly adhesive and self-healing γ-PGA/PEDOT:PSS conductive hydrogels enabled by multiple hydrogen bonding for wearable electronics. *Nano Energy* **95**, 106991 (2022).

S13. Zhang Z, Chen G, Xue Y, Duan Q, Liang X, Lin T, *et al.* Fatigue-resistant conducting polymer hydrogels as strain sensor for underwater robotics. *Adv. Funct. Mater.* **33**, 2305705 (2023).

S14. Kim S, Choi H, Son D, Shin M. Conductive and adhesive granular alginate hydrogels for on-tissue writable bioelectronics. *Gels* **9**, 167 (2023).

S15. Zhu D, Miao M, Du X, Peng Y, Wang Z, Liu S, *et al.* Long/short chain crosslinkers-optimized and PEDOT:PSS-enhanced covalent double network hydrogels rapidly prepared under green LED irradiation as flexible strain sensor. *Eur. Polym. J.* **174**, 111327 (2022).

S16. Roshanbinfar K, Vogt L, Greber B, Diecke S, Boccaccini AR, Scheibel T, *et al.* Electroconductive biohybrid hydrogel for enhanced maturation and beating properties of engineered cardiac tissues. *Adv. Funct. Mater.* **28**, 1803951 (2018).

S17. Gao Q, Li C, Wang M, Zhu J, Gao C. A low-hysteresis, self-adhesive and conductive PAA/PEDOT:PSS hydrogel enabled body-conformable electronics. *J. Mater. Chem. C* **11**, 9355-9365 (2023).

S18. Zeng M-Z, Wei D, Ding J, Tian Y, Wu X-Y, Chen Z-H, *et al.* Dopamine induced multiple bonding in hyaluronic acid network to construct particle-free conductive hydrogel for reliable electro-biosensing. *Carbohydr. Polym.* **302**, 120403 (2023).

S19. Gong J-Y, Sun F-C, Pan Y-C, Fei A-M, Leicheng S-F, Du F-P, *et al.* Stretchable and tough PAANa/PEDOT:PSS/PVA conductive hydrogels for flexible strain sensors. *Mater. Today Commun.* **33**, 104324 (2022).

S20. Prameswati A, Nurmaulia Entifar SA, Han JW, Wibowo AF, Kim JH, Sembiring YSb, *et al.* Self-healable conductive hydrogels with high stretchability and ultralow hysteresis for soft electronics. *ACS Appl. Mater. Interfaces* **15**, 24648-24657 (2023).

S21. Testore D, Zoso A, Kortaberria G, Sangermano M, Chiono V. Electroconductive photo-curable PEGDA-gelatin/PEDOT:PSS hydrogels for prospective cardiac tissue engineering

application. *Front. Bioeng. Biotechnol.* **10**, 897575 (2022).

S22. Bian Z, Li Y, Sun H, Shi M, Zheng Y, Liu H, *et al.* Transparent, intrinsically stretchable cellulose nanofiber-mediated conductive hydrogel for strain and humidity sensing. *Carbohydr. Polym.* **301**, 120300 (2023).

S23. Zhang Y, Zhang M, Zhang R, Liu H, Chen H, Zhang X, *et al.* Conductive GelMA/PEDOT: PSS hybrid hydrogel as a neural stem cell niche for treating cerebral ischemia-reperfusion injury. *Frontiers in Materials* **9**, 914994 (2022).

S24. Xu L, Liu S, Zhu L, Liu Y, Li N, Shi X, *et al.* Hydroxypropyl methyl cellulose reinforced conducting polymer hydrogels with ultra-stretchability and low hysteresis as highly sensitive strain sensors for wearable health monitoring. *Int. J. Biol. Macromol.* **236**, 123956 (2023).

S25. Kasimu A, Zhu H, Meng Z, Qiu Z, Wang Y, Li D, *et al.* Development of electro-conductive composite bioinks for electrohydrodynamic bioprinting with microscale resolution. *Adv. Biol.* **7**, 2300056 (2023).

S26. Furlani F, Montanari M, Sangiorgi N, Saracino E, Campodoni E, Sanson A, *et al.* Electroconductive and injectable hydrogels based on gelatin and PEDOT:PSS for a minimally invasive approach in nervous tissue regeneration. *Biomater. Sci.* **10**, 2040-2053 (2022).

S27. Zhang M, Wang Y, Liu K, Liu Y, Xu T, Du H, *et al.* Strong, conductive, and freezing-tolerant polyacrylamide/PEDOT:PSS/cellulose nanofibrils hydrogels for wearable strain sensors. *Carbohydr. Polym.* **305**, 120567 (2023).

S28. Casella A, Panitch A, Leach JK. Electroconductive agarose hydrogels modulate mesenchymal stromal cell adhesion and spreading through protein adsorption. *J. Biomed. Mater. Res. Part A* **111**, 596-608 (2023).
