# Supplementary material for: Boosting hydrogel conductivity via water-dispersible conducting polymers for injectable bioelectronics
Source: Nat Commun. 2025 Apr 22;16:3755. doi: 10.1038/s41467-025-59045-1 (PMC12015517; doi:10.1038/s41467-025-59045-1)
Supplement: Supplementary file 2 — Reporting Summary [file 41467_2025_59045_MOESM2_ESM.pdf]

nature portfolio

Corresponding author(s): Wei Gao

Last updated by author(s): Mar 31, 2025

Reporting Summary

Nature Portfolio wishes to improve the reproducibility of the work that we publish. This form provides structure for consistency and transparency in reporting. For further information on Nature Portfolio policies, see our Editorial Policies and the Editorial Policy Checklist.

Statistics

For all statistical analyses, confirm that the following items are present in the figure legend, table legend, main text, or Methods section.

n/a	Confirmed	
☐	☒	The exact sample size (n) for each experimental group/condition, given as a discrete number and unit of measurement
☐	☒	A statement on whether measurements were taken from distinct samples or whether the same sample was measured repeatedly
☐	☒	The statistical test(s) used AND whether they are one- or two-sided<br>*Only common tests should be described solely by name; describe more complex techniques in the Methods section.*
☒	☐	A description of all covariates tested
☐	☒	A description of any assumptions or corrections, such as tests of normality and adjustment for multiple comparisons
☐	☒	A full description of the statistical parameters including central tendency (e.g. means) or other basic estimates (e.g. regression coefficient) AND variation (e.g. standard deviation) or associated estimates of uncertainty (e.g. confidence intervals)
☐	☒	For null hypothesis testing, the test statistic (e.g. F, t, r) with confidence intervals, effect sizes, degrees of freedom and P value noted<br>*Give P values as exact values whenever suitable.*
☒	☐	For Bayesian analysis, information on the choice of priors and Markov chain Monte Carlo settings
☒	☐	For hierarchical and complex designs, identification of the appropriate level for tests and full reporting of outcomes
☒	☐	Estimates of effect sizes (e.g. Cohen's d, Pearson's r), indicating how they were calculated

Our web collection on statistics for biologists contains articles on many of the points above.

Software and code

Policy information about availability of computer code

Data collection	BlueHill v3 was used to collect mechanical testing data. Rheological data was obtained by RheCompass v1.3. A Python code was used for 3D printing G-Code generation (available at https://github.com/hmontazerian/DXF-to-GCode.git).
Data analysis	GraphPad Prism v10 was used for statistical analyses. ImageJ v2.1.0 was used to analyze fluorescent microscope images. Gamry Echem Analyst 2 was used to analyze the EIS data. Matlab codes used for low-pass filtering of the biosignal data (available at https://github.com/hmontazerian/biopotential-signals-recording.git).

For manuscripts utilizing custom algorithms or software that are central to the research but not yet described in published literature, software must be made available to editors and reviewers. We strongly encourage code deposition in a community repository (e.g. GitHub). See the Nature Portfolio guidelines for submitting code & software for further information.

Data

Policy information about availability of data

All manuscripts must include a data availability statement. This statement should provide the following information, where applicable:

- Accession codes, unique identifiers, or web links for publicly available datasets
- A description of any restrictions on data availability
- For clinical datasets or third party data, please ensure that the statement adheres to our policy

Data source is provided with this manuscript. All raw and analysed datasets generated during the study are available from the corresponding author on request.

Research involving human participants, their data, or biological material

Policy information about studies with human participants or human data. See also policy information about sex, gender (identity/presentation), and sexual orientation and race, ethnicity and racism.

Reporting on sex and gender	N/A
Reporting on race, ethnicity, or other socially relevant groupings	N/A
Population characteristics	N/A
Recruitment	N/A
Ethics oversight	N/A

Note that full information on the approval of the study protocol must also be provided in the manuscript.

Field-specific reporting

Please select the one below that is the best fit for your research. If you are not sure, read the appropriate sections before making your selection.

☒ Life sciences ☐ Behavioural & social sciences ☐ Ecological, evolutionary & environmental sciences

For a reference copy of the document with all sections, see nature.com/documents/nr-reporting-summary-flat.pdf

Life sciences study design

All studies must disclose on these points even when the disclosure is negative.

Sample size	Sample sizes were determined based on standard practices in the field, considering experimental feasibility, biological variability, and statistical power requirements. For all in vitro experiments, including hemostatic absorbance measurements, a minimum of triplicates (n = 3) was used to ensure reproducibility and account for technical variability. For animal studies, sample sizes were chosen based on previous literature and the expected effect size, with n = 4 for hemostatic absorbance measurements and n = 5 for general animal studies. For in vivo immunostaining, n = 6 was used to account for potential biological variability and enhance statistical robustness. The chosen sample sizes align with similar studies in the field and have been sufficient to observe statistically significant differences where applicable. Data variability was assessed post hoc, and statistical analyses were performed accordingly.
Data exclusions	No data was excluded.
Replication	Minimum of three independent samples were replicated for each experiment.
Randomization	Data represent randomly allocated groups.
Blinding	Blinding was not utilized during data acquisition and analysis because experiments did not introduce subjective bias. The outcomes were determined using predefined, objective metrics, minimizing the risk of operator influence.

Reporting for specific materials, systems and methods

We require information from authors about some types of materials, experimental systems and methods used in many studies. Here, indicate whether each material, system or method listed is relevant to your study. If you are not sure if a list item applies to your research, read the appropriate section before selecting a response.

Materials & experimental systems

n/a	Involved in the study	
☐	☒	Antibodies
☒	☐	Eukaryotic cell lines
☒	☐	Palaeontology and archaeology
☐	☒	Animals and other organisms
☒	☐	Clinical data
☒	☐	Dual use research of concern
☒	☐	Plants

Methods

n/a	Involved in the study	
☒	☐	ChIP-seq
☐	☒	Flow cytometry
☒	☐	MRI-based neuroimaging

Antibodies

Antibodies used	CD3 (BioLegend, catalog Number: cat 100205, lot #B424757, clone 17A2);<br>Ly6G (BioLegend, catalog Number: 127607, lot #B440678, clone 1A8);<br>F4/80 (BioRad, catalog Number: MCA497A488, lot #18567, clone #Cl:A3-1);<br>CD80 (BioLegend, catalog Number: cat: 600055, lot #b444189, clone W17200C)
Validation	Each antibody used in this study was validated based on the manufacturer's data available on their website, including specificity and performance under relevant conditions.<br>F4/80: https://www.bio-rad-antibodies.com/monoclonal/mouse-f4-80-antibody-cl-a3-1-mca497.html?f=purified<br>CD3: https://www.biolegend.com/en-us/products/pe-anti-mouse-cd3-antibody-47<br>CD80: https://www.biolegend.com/en-us/products/pe-anti-mouse-cd80-antibody-26489<br>Ly6G: https://www.biolegend.com/en-us/products/pe-anti-mouse-ly-6g-antibody-4777

Animals and other research organisms

Policy information about studies involving animals; ARRIVE guidelines recommended for reporting animal research, and Sex and Gender in Research

Laboratory animals	Ob/ob mice, B6.Cg-Lepob/J mice (The Jackson Laboratory, Bar Harbor, ME, USA) for wound monitoring tests and black male C57BL/6 mice, 6-8 weeks old, obtained from The Jackson Laboratory in the USA for biocompatibility studies housed in standard laboratory conditions. Mice were housed in standard laboratory conditions with a 12-hour light/dark cycle, ambient temperature maintained at 25 ± 2°C, and relative humidity of 50 ± 10%. Animals were provided ad libitum access to laboratory pellets and purified water in pathogen-free facilities.
Wild animals	This study did not involve wild animals.
Reporting on sex	Gender was not considered as a factor that would affect biocompatibility, immune response, and wound healing response to the developed materials.
Field-collected samples	This study did not include samples collected from the field.
Ethics oversight	Lundquist Institute (#22747-01); Protocol no. IA23-1800 at California Institute of Technology

Note that full information on the approval of the study protocol must also be provided in the manuscript.

Plants

Seed stocks	N/A
Novel plant genotypes	N/A
Authentication	N/A

Flow Cytometry

Plots

Confirm that:

☒ The axis labels state the marker and fluorochrome used (e.g. CD4-FITC).

☒ The axis scales are clearly visible. Include numbers along axes only for bottom left plot of group (a 'group' is an analysis of identical markers).

☒ All plots are contour plots with outliers or pseudocolor plots.

☒ A numerical value for number of cells or percentage (with statistics) is provided.

Methodology

Sample preparation	Bone marrow-derived macrophages (BMDMs), isolated from the tibia and femur bones of 5-6 week old 5C5BL/6 mice were exposed to the material extracts.
Instrument	Attune, ThermoFisher Scientific, USA

Software	Attune NxT software
Cell population abundance	The abundance of relevant cell populations within post-sort features was determined by flow cytometry analysis of stained macrophages. Cell purity was assessed by re-analyzing a subset of the sorted cells, with purity calculated based on the percentage of F4/80+ and CD80+ cells relative to total events. Purity typically exceeded 95%, as determined by gating on the F4/80+ population and confirmed by isotype controls.
Gating strategy	The abundance of relevant cell populations within post-sort features was determined by flow cytometry analysis of stained macrophages. Cell purity was assessed by re-analyzing a subset of the sorted cells, with purity calculated based on the percentage of F4/80+ and CD80+ cells relative to total events. Purity typically exceeded 95%, as determined by gating on the F4/80+ population and confirmed by isotype controls.

☐ Tick this box to confirm that a figure exemplifying the gating strategy is provided in the Supplementary Information.
